# Supplementary material for: Happy or healthy? How members of the public prioritise farm animal health and natural behaviours
Source: PLoS One. 2021 Mar 3;16(3):e0247788. doi: 10.1371/journal.pone.0247788 (PMC7928445; doi:10.1371/journal.pone.0247788)
Supplement: S1 File — (DOCX) [file pone.0247788.s001.docx]

# SECTION 1: Demographic Factors

**1. How would you describe yourself?**

Male

Female

In another way

Prefer not to say

**2. In what year where you born?** (Please input 0, if you would prefer not to say)

|  |
| --- |

**3. What is the highest level of education you have completed** (If you’re currently still in education, please indicate the highest level you have received)**?**

Primary

Secondary

Undergraduate Degree

Post-graduate Degree

Other (Please specify): ______________________________

Prefer not to say

**4. What is the annual income of your household?** (Annual household income is the combined gross pay, before tax, of all the income-contributing people in your home).

Less than £20,000

£20,000 to £34,999

£35,000 to £49,999

£50,000 to £74,999

£75,000 to £99,999

Over £100,000

Prefer not to say

**5. Which of the following best describes your current dietary** **preferences?**

Meat is a regular part of my diet

I am flexitarian (I eat meat some of the time)

I am vegetarian

I am vegan

I am pescatarian (I eat fist but not meat)

Prefer not to say

Other (please specify): _________________________________

**6. How would you describe the area in which you live?**

Urban (e.g. city, large town)

Suburban

Semi-rural (e.g. village, slightly rural area)

Rural

Prefer not to say

**7. What is the first part of your postcode? (e.g. EH3 or DG16)** (This will be used only to determine your broad geographical region) **(Please input 0 if you would** **prefer not to say)**

|  |
| --- |

# Section 2: Factorial Vignette Scenario

**Please read carefully and consider the following scenario, in the box below, where a livestock farmer describes their approach to managing and caring for the health and natural behaviours of the animals on their farm:**

*(Participants were randomly assigned ONE of the following scenarios)*

| "I want my animals to be healthy. To me, this means having them stress free, pain free and injury free, whilst also being aware of any health issues that might be arising and dealing with them.  At the same time, I don’t think I need to do anything specific to support natural behavioural expression in my animals" |
| --- |
| "I want my animals to be healthy. To me, this means having them stress free, pain free and injury free, whilst also being aware of any health issues that might be arising and dealing with them.  At the same time, I want my animals to be able to express their natural behaviours. So, I try to make sure that they can go and have a wander around and see their surroundings, they can choose the animals they want to be around, lie down where they want to lie down and eat when they want to eat" |
| "When it comes to health, I am inclined to let nature take its course. I’d rather let the animal look after itself than intervene. For example, if I see the odd animal with a sore foot, I’ll leave it alone and let it heal in its own time.  At the same time, I don’t think I need to do anything specific to support natural behavioural expression in my animals" |
| "When it comes to health, I am inclined to let nature take its course. I’d rather let the animal look after itself than intervene. For example, if I see the odd animal with a sore foot, I’ll leave it alone and let it heal in its own time.  At the same time, I want my animals to be able to express their natural behaviours. So, I try to make sure that they can go and have a wander around and see their surroundings, they can choose the animals they want to be around, lie down where they want to lie down and eat when they want to eat" |

(NOTE: Health can be taken to mean the absence or minimisation of illness, disease and physical discomfort. Natural behavioural expression refers to behaviours that animals are internally motivated to exhibit and behaviours that are pleasurable)

**Now please answer the following questions in relation to the information provided in the above scenario:**

**1. How would you rate the overall well-being of the animals on this farm?** (drag the sliding scale to any number from 0 to 10 to indicate your response)


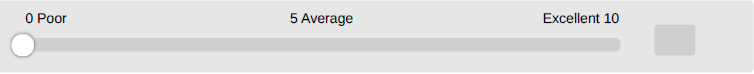


**Why did you give this rating for overall well-being? Please describe in your own words below:**

|  |
| --- |

**2. Did you think of a particular type of farm animal when you read the above scenario?**

No

Yes

If Yes: Please describe here:

|  |
| --- |

**3. How would you rate the overall physical health of the animals on this farm?** (drag the sliding scale to any number from 0 to 10 to indicate your response)


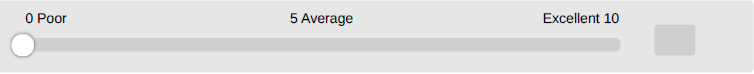


**4. How would you rate the overall mental health of the animals on this farm?** (drag the sliding scale to any number from 0 to 10 to indicate your response)


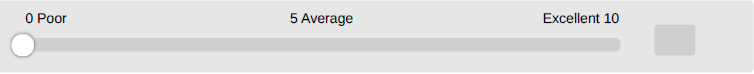


**5. How would you rate the overall ability of the animals on this farm to be productive? (i.e. their ability to be able to produce desired outputs such as milk or meat)** (drag the sliding scale to any number from 0 to 10 to indicate your response)


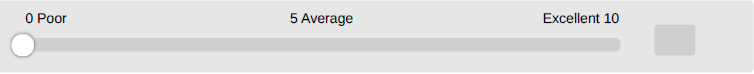


**6. How do you think other members of the public would rate the well-being of the animals on this farm?** (drag the sliding scale to any number from 0 to 10 to indicate your response)


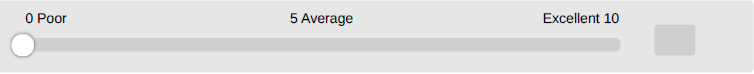


**7. If you were to change anything about this farm; what would that be?**

|  |
| --- |

Section 3: Attitudes to the importance of minimising health issues and promoting natural behaviours

When it comes to caring for the well-being of farm animals, how important do you think the following are:

(NOTE: Health can be taken to mean the absence or minimisation of illness, disease and physical discomfort. Natural behavioural expression refers to behaviours that animals are internally motivated to exhibit and behaviours that are pleasurable)

## Minimising Health issues


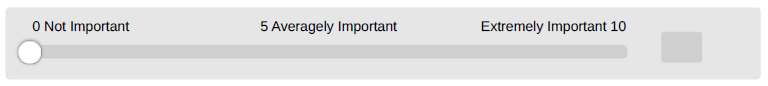


## Promoting the expression of natural behaviours


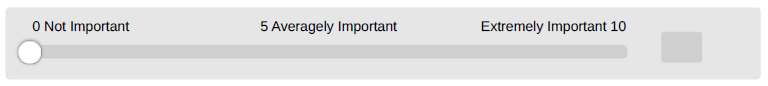


**When it comes to caring for the well-being of farm animals, which of the following do you think is the most important? (please select one only)**

Minimising health issues

Promoting the expression of natural behaviours

# Section 4: Belief in Animal Mind Scale

**Please rate, from 0 to 10, the extent to which you agree or disagree with the following statements?**

**1. Most farm animals are unaware of what is happening to them**


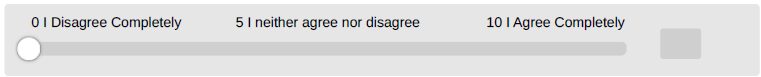


**2. Most farm animals are capable of experiencing a range of feelings and emotions (e.g. pain, fear, contentment, maternal affection)**


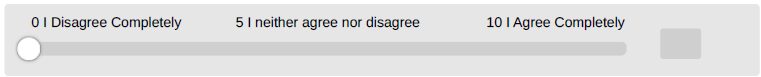


**3. Most farm animals are able to think to some extent to solve problems and make decisions about what to do**


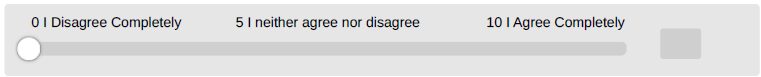


**4. Most farm animals are more like computer programs, i.e. mechanically responding to instinctive urges without awareness of what they are doing.**


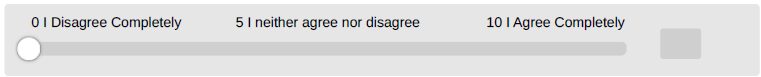


# Section 5: Participant experience of farming

**Which of the following best describes your experience of livestock farming** (i.e. farms which keep pigs, sheep, cows, goats or poultry for the purposes of food production)?:

I have no experience with farm animals or livestock farms

I grew up on a livestock farm

I have relatives or friends who are livestock farmers

I have been to a livestock farm(s) on an educational visit (i.e. on a school trip, to a farm open-day or to an educational farm)

I have worked on a livestock farm

Other (please specify):_______________________________________
