# Supplementary material for: Happy or healthy? How members of the public prioritise farm animal health and natural behaviours
Source: PLoS One. 2021 Mar 3;16(3):e0247788. doi: 10.1371/journal.pone.0247788 (PMC7928445; doi:10.1371/journal.pone.0247788)
Supplement: Attachment — Submitted filename: Happy or healthy REVIEW.docx [file pone.0247788.s003.docx]

**Happy or Healthy? How members of the public prioritise farm animal health and natural behaviours**

**Authors: Belinda Vigors, David A. Ewing and Alistair B. Lawrence**

A really interesting and well written paper on an important and timely subject. The investigation was introduced well, the design of the study was thorough and suitable for the aim and objectives of the research, and the findings discussed in-depth. I am happy to recommend this manuscript for publication after addressing the following minor revisions.

- What is natural/naturalness? Described by the authors as related to “normality” (**line 52**), but what does this mean? The authors make clear what unnatural and non-normal factors are (e.g., **lines 54 – 56**) but not the opposite, or what natural factors may be. The authors do acknowledge that naturalness is difficult to define (**line 70**), and I agree it is, but some text on what this might be/what the existing definitions are would be useful, especially as the study examines how the public value naturalness and the promotion of natural behaviours in farm animals.
- Who are the “key stakeholders”? (**Lines 69, 93, etc**.). A list of potential, or actual key stakeholders would be useful here.
- The use of vignettes is clearly explained, however the vignette names (high health, low health, high behaviour, low behaviour) is not clearly linked to these explanations until the end of the paragraph (**lines 157 – 159**). A very minor edit moving these descriptions to earlier in the text would be helpful to the reader.
- **Lines 164 – 165 –** do you give descriptors for each of the numeric values in the scale, or just the mid-point (5) and the extreme values (0 and 10)? Again, this is a very minor point, but would help the reader understand the survey method.
- **Lines 245 – 257 –** Examples of sentiment, i.e., positive, negative and neutral language would be useful here. E.g., “Language categorised as positive/negative/neutral sentiment included …….”. Was this done manually, or using computer software?
- A point to bring up in the introduction **(Lines 39 onwards)** and discussion **(Lines 635 onwards)** – why survey the views of the UK public specifically, why not a sample of publics from another country? I think a strong case could be made in the introduction and then discussed later as to the importance of understanding public opinion in relation to the UK leaving the EU and the Common Agricultural Policy and therefore changes in the way that agriculture in the UK will be funded, e.g., public money for public goods, and animal health and welfare being described in policy documents as a public good (i.e., the Animal Health and Welfare Pathway). This would highlight how timely and important this piece of research is.
