# Supplementary material for: Happy or healthy? How members of the public prioritise farm animal health and natural behaviours
Source: PLoS One. 2021 Mar 3;16(3):e0247788. doi: 10.1371/journal.pone.0247788 (PMC7928445; doi:10.1371/journal.pone.0247788)
Supplement: Attachment — Submitted filename: Response to Reviewers.docx [file pone.0247788.s005.docx]

Dr. Belinda Vigors

Animal and Veterinary Sciences,

SRUC,

West Mains Road,

Edinburgh,

EH3 9RG,

UK

Resubmission date: 11/02/21

Dear Editor,

Please find attached our revised submission to *PLOS ONE* for the paper “Happy or Healthy? How members of the public prioritise farm animal health and natural behaviours**”.** Thank you for your consideration of this paper and for the positive feedback on our first review.

In taking on board your recommendations, regarding the need to address the potential limitations of the factorial design highlighted by reviewer 1, we have made an addition to the discussion section of the paper. Here, we outline the reasons behind the randomising of the vignettes between participants and address how this was dealt with in the analysis. This addition can be seen in the tracked changes manuscript from line 835 onwards.

I look forward to hearing from you in due course.

Sincerely,

Belinda Vigors

Corresponding Author.
